# Machine Learning-Based Prediction of Cattle Activity Using Sensor-Based Data

**DOI:** 10.3390/s24103157

**Published:** 2024-05-16

**Authors:** Guillermo Hernández, Carlos González-Sánchez, Angélica González-Arrieta, Guillermo Sánchez-Brizuela, Juan-Carlos Fraile

**Affiliations:** 1 Grupo de Investigación BISITE, Universidad de Salamanca, 37008 Salamanca, Spain; guillehg@usal.es (G.H.); angelica@usal.es (A.G.-A.); 2 ITAP (Instituto de las Tecnologías Avanzadas de la Producción), Universidad de Valladolid, 47011 Valladolid, Spain; carlos.gonzalezs@estudiantes.uva.es (C.G.-S.); guillesanbri@gmail.com (G.S.-B.)

**Keywords:** cow, extensive livestock, machine learning, monitoring, sensorized wearable device

## Abstract

Livestock monitoring is a task traditionally carried out through direct observation by experienced caretakers. By analyzing its behavior, it is possible to predict to a certain degree events that require human action, such as calving. However, this continuous monitoring is in many cases not feasible. In this work, we propose, develop and evaluate the accuracy of intelligent algorithms that operate on data obtained by low-cost sensors to determine the state of the animal in the terms used by the caregivers (grazing, ruminating, walking, etc.). The best results have been obtained using aggregations and averages of the time series with support vector classifiers and tree-based ensembles, reaching accuracies of 57% for the general behavior problem (4 classes) and 85% for the standing behavior problem (2 classes). This is a preliminary step to the realization of event-specific predictions.

## 1. Introduction

The Food and Agriculture Organization (FAO) lists a series of challenges that the world is currently facing, and which will be aggravated in the future: population growth generates an increase in demand for agricultural products, while urbanization shifts labor from the food and agricultural systems typical of more rural areas into cities [1]. Simultaneously, meat consumption trends are increasing globally, while modern societies are growingly concerned about food safety and quality, ethical treatment and well-being of farm animals and the minimization of the environmental impact of livestock production [2]. Farms are facing increasing economic pressure for optimization and cost reduction, leading to an increment in the number of animals per farm (which favors economies of scale) and reducing labor costs associated with expert farmers who observe, monitor, and manage the herd [3].

These challenges especially affect very extensive livestock farming systems, which are typical to Spain’s *dehesas* and Portuguese *montados*, characterized by a sustainable, much lower-intensive use of land than other European systems also considered extensive [4]. The increased distance to and between the animals requires more time and fuel to monitor, which translates into increased costs and reduced competitiveness [5].

To address these issues, the use of automation has been steadily growing in the field of farming, leading to the development of Precision Livestock Farming (PLF), a management system of production based on new technologies which aims to improve the efficient use of resources and to reduce costs. Recent advances in the Wireless Internet of Things (W-IoT) and wearable technology allow, among others, the development of PLF techniques based on remote monitoring of animal health and welfare parameters in a continuous and automated way [6].

Accelerometers are one of the most common methods used to monitor cattle activity. Data from these sensors have been used to classify physiological states such as walking, ruminating, lying, etc., [7]. One of the best indicators used to predict calving is the frequency of lying bouts (transitions from standing to lying) [8]. Statistical analysis can be used to detect deviation from normal cow behavior, which can be used as an important input for the farmer [8].

The detection of lying bouts is trivial using sensors placed on the legs or back of an animal [9,10]. Collars [11,12] and ear-based devices [13,14] on the other hand, make it easier to detect feeding events (eating, drinking, ruminating), but the differentiation between lying and standing behaviours is challenging, due to the similar characteristics of the signals measured by the sensors [15]. Our focus on long-term monitoring, however, limits the possible location of the system to the collar, due to the size and weight of the batteries.

Although many prototypes have been presented in different studies, none combines the Global Navigation Satellite System (GNSS) reception, wireless capabilities, and battery energy management allowing long-term monitoring of our design, which has been optimized for use in extensive farming. The proposed design will allow the collection, analysis, and decision-making based on real-time data obtained from the studied animals.

In this study, data compiled by a low-cost neck-mounted sensorized wearable device, presented in González-Sánchez et al. [16] is analyzed, and different machine learning algorithms are used to produce models that allow the detection of different physiological states of the animal based on the information captured by the sensors.

## 2. Materials and Methods

In this section, we present the equipment used during this study, as well as the data collection procedure and the structure of the resulting dataset.

### 2.1. Hardware

To collect the data that has been used to train and evaluate the different machine learning architectures presented in this paper, we have fitted neck-mounted wearable devices, previously presented in González-Sánchez et al. [16], to different cows. The collars are powered using a lithium-ion battery and enclose the following components in a polymer (ABS) box sealed with a nitrile O-ring:

- Digital thermometer in contact with the skin of the cows that provides temperature data in a −55 to 125 °C range with an accuracy of ±0.5 °C.
- GNSS sensor that supplies localization data (latitude, longitude, and altitude) with a 2.5 m CEP.
- 9-axis Inertial Measurement Unit (IMU) which encompasses an accelerometer (3-axis), a gyroscope (3-axis), and a magnetometer (3-axis).
- Embedded microcontroller dedicated to managing and storing the readings from the previous sensors into an SD card.

### 2.2. Data Collection

The data collection procedure has been carried out on an extensive livestock farm in Carrascal de Barregas, Salamanca, Spain consistently for a year, starting in August 2020 and ending in August 2021. This way, the recorded data points were evenly distributed in the yearly seasonal cycle. As previously mentioned, the collars have been used to collect sensor data associated with the physical behavior of cattle. This data has been sampled at a frequency of 1 Hz in the GNSS and temperature sensor, while the IMU was configured to output its reading at a 17.6 Hz rate. Furthermore, two human observers directly monitored and recorded the actions performed by the cows using a custom-made PC software application running on a laptop. Further information on the procedure is available in González-Sánchez et al. [16]. The annotation of the cows’ actions was separated into two groups of labels: general behavior and standing behavior. As part of the actions within these two groups overlap, only one of the groups was used at a time. The labels that compose each group are presented in Table 1.

**Figure 1:**
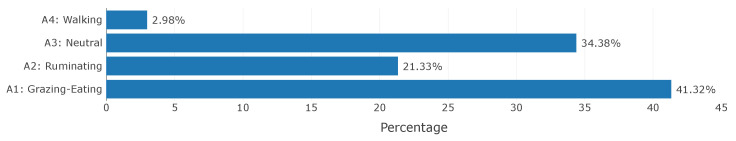
General behaviour class distribution in the dataset.

**Figure 2:**
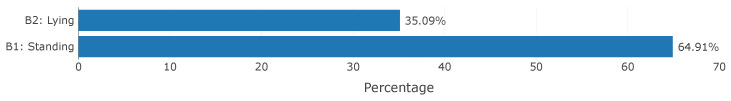
Standing behaviour class distribution in the dataset.

On a weekly basis, the recorded readings from the sensors were extracted from the collars. Once the data was successfully collected, the human-annotated behavior of each cow was cross-matched with the sensor data based on the timestamp of the reading to annotate each registry, forming the final dataset. The distribution of the annotations in each group present in the final dataset is presented in Figure 1 and Figure 2.

There is a slight unbalance between classes in both groups, especially in action A4 (walking), which is only present in a 3% of the labels corresponding to the general behavior group.

The dataset obtained from the collection procedure contains more than 1900 h of annotated data from 7 different specimens of beef cattle. As part of the sensor installation works at 17.6 Hz, the dataset is composed of more than 120 million labeled data points. The consequent abundance of data ensures a varied data sample, allowing us to implement an independent cross-validation procedure through the utilization of data from cows that have been isolated (not used) during the fitting process of the models to evaluate the quality of the results.

### 2.3. Data Preprocessing

The data can be considered as a set of non-consecutive equispaced time series for each animal. The sampling frequency is too small to attempt predictions at that level, which makes it necessary to group the data in some time intervals that constitute the prediction unit of the system.

Thus, the first step will consist of the aggregation in moving time windows of a width that we will consider variable in the study, but that has to represent a compromise between the prediction capacity—it will represent the time range for which a prediction will be valid—and the computational capabilities of the system.

As aggregation measures for time series of the type ${\left(x_{n}\right)}_{i=1}^{n}$ we have considered the moments of different order of the distribution: the mean

(1) $$\bar{x}=\frac{1}{n}\sum_{i=1}^{n}x_{i},$$

the standard deviation

(2) $$\sigma_{x}=\sqrt{\frac{1}{n-1}\sum_{i=1}^{n}{\left(x_{i}-\mu \right)}^{2}},$$

and the skewness, calculated using the unbiased Fisher-Pearson standardized moment coefficient, which is given by

(3) $$G_{1}\left(x\right)=\frac{\sqrt{n(n-1)}}{n-2}\frac{m_{3}\left(x\right)}{m_{2}^{3/2}\left(x\right)},$$

where

(4) $$m_{k}\left(x\right)=\frac{1}{n}\sum_{i=1}^{n}{\left(x-x_{i}\right)}^{k},$$

is the biased sample *k*-th central moment.

Class labels are always aggregated using the mode, given that they are discrete attributes.

### 2.4. Model Construction

**Figure 3:**
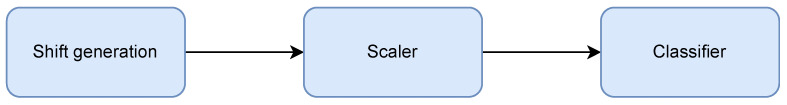
Stages in the construction of models.

The stages in model building are shown schematically in Figure 3. The first of these stages is the generation of shifted temporal information using the preceding values in chronological order. In case no information is available, these instances are discarded. Thus, a system using *k* groups of the preceding time steps, each of them obtained by aggregation in an interval δt, will be required to run the amount of time corresponding to these before it starts predicting, which accounts for kδt units of time.

The second stage shown in Figure 3 is standardization. Several of the classifiers to be described later are scale-dependent, so it is necessary to define some prior standardization or normalization process. Each of the attributes has been standardized by subtracting the mean and dividing by the standard deviation, that is,

(5) $$x_{\mathrm{scaled}}=\frac{x-\bar{x}}{\sigma_{x}}.$$

The final stage is the training of classifier algorithms based on machine learning implementations available in the scikit-learn package [17], as shown in Table 2. The resulting methods include a trivial classifier that predicts the majority class to serve as a baseline, logistic regression, support vector regression, decision trees, and ensemble methods based on them (random forest, gradient boosting, and extremely randomized trees).

### 2.5. Evaluation

To design the evaluation procedure, we seek a process that adequately represents the generalization capacity of the proposal. To this end, we seek to study the behavior of the system when applied to a new cow. To perform this evaluation in an unbiased manner, we train a model with all but one of the cows, The system’s performance would be evaluated then with this excluded data. The process would then be repeated by varying the subset of data that is left out so that an unbiased evaluation is available for each of the data in the set. This methodology is called Leave One Group Out cross-validation.

Since two different types of annotations were performed (Figure 1 and Figure 2) two evaluations will be performed, one for each of these scenarios. Accuracy has been chosen as a global metric to compare the models. Other metrics will be analyzed for more relevant models, including $F_{1}$, precision, recall, Cohen’s κ, and Matthews’ φ.

## 3. Results and Discussion

**Figure 4:**
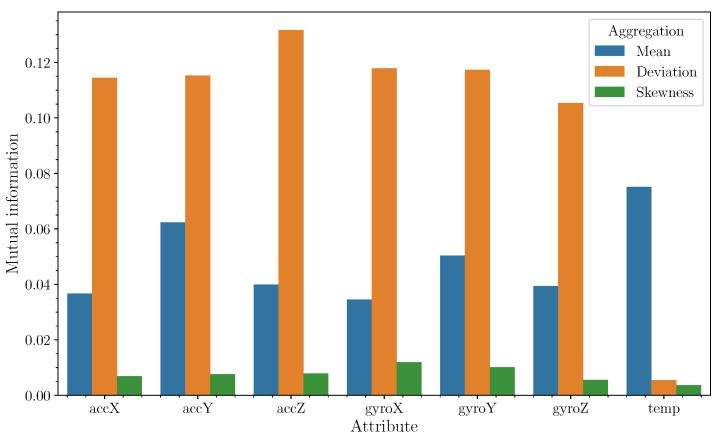
Mutual information with respect to class (*y*-axis) for the different variables (*x*-axis) as a function of aggregation methods. Labels accX, accY, accZ refer to triaxial acceleration; gyroX, gyroY and gyroZ refer to triaxial angular velocity. temp represents the measured temperature. Data was obtained from a random sample of 200,000 values.

Figure 4 displays the mutual information metrics for various aggregation methods in relation to the class. It is evident that, for dynamic components such as angular velocity and acceleration, the aggregation mechanism yielding the highest mutual information is the standard deviation. This implies that standard deviation captures more about the underlying patterns of these dynamic components with respect to the class than other aggregation methods. Conversely, the third-order moment (skewness) contributes minimal information in this context, indicating that its utility for distinguishing between classes is limited. Based on these findings, it is advisable to limit the aggregation to second-order moments.

Regarding temperature data, a different trend is observed. The mean temperature value provides more informative insights into class differentiation than the standard deviation. This suggests that the average temperature over time is more indicative of the class than the variability of temperature readings. Therefore, for temperature data, focusing on the mean value as the primary aggregation metric could be more beneficial for classification purposes.

**Figure 5:**
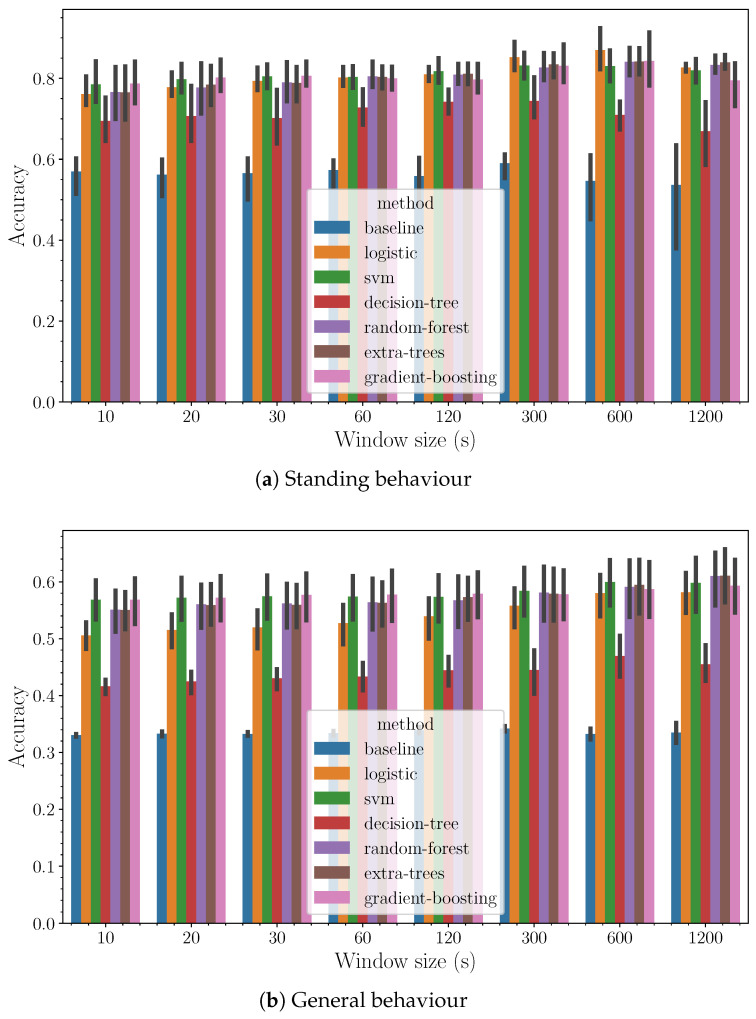
Accuracy (*y*-axis) as a function of the method (hue) and the size of the window (*x*-axis).

The accuracy outcomes for each of the evaluated algorithms are illustrated in Figure 5. Here, a pattern emerges showing a slight improvement in accuracy as the window size increases. Although the increment is minimal, it conveys an essential insight into the selection of an optimal step size. This step size should align with the practical timeframe for human actions, particularly concerning the interval within which decisions are made based on the evolving situation. A pragmatic approach would be to choose a window duration that supports timely and effective decision-making, without unnecessarily complicating the analysis with too granular data or overlooking important trends due to overly broad windows. Therefore, based on the observed data and considering the dynamics of human response times, a window size ranging from 30 to 120 s emerges as a reasonable compromise. This is also consistent with the uncertainty present in the annotation process itself, subject to possible delays between observation and annotation. The proposed time scale is consistent with the labelers’ perception, as has been corroborated through interviews with them.

In evaluating the performance of the various algorithms, it becomes clear that a subset of them stands out in terms of efficacy. This group, distinguished by its superior results, comprises ensemble methods such as random forest, gradient boosting, and extra randomized trees, alongside support vector machines (SVMs).

**Figure 6:**
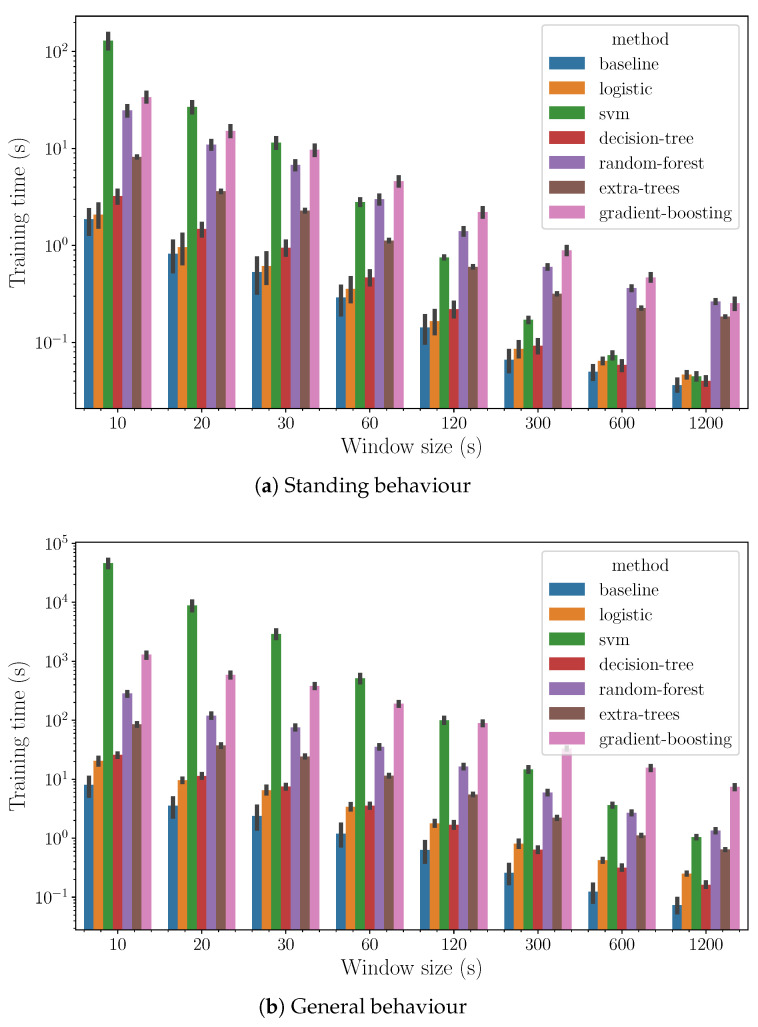
Computing time (*y*-axis) as a function of the method (hue) and the size of the window (*x*-axis).

In Figure 6, we observe the computational costs of the algorithms on a semilogarithmic scale, as introduced in Figure 5a. Among the top performers in accuracy, extremely randomized trees excel in computational efficiency for small window sizes, while support vector machines are more efficient for larger window sizes. This distinction highlights the need to balance accuracy with computational resources when choosing an algorithm. Essentially, for quick, small-scale analyses, extremely randomized trees are preferred due to their speed and lower computational demands. In contrast, for more extensive data analyses, support vector machines are better suited because they handle increased data volume efficiently. This insight guides a strategic selection of algorithms based on the analysis scale and computational constraints, ensuring optimal performance and efficiency.

**Figure 7:**
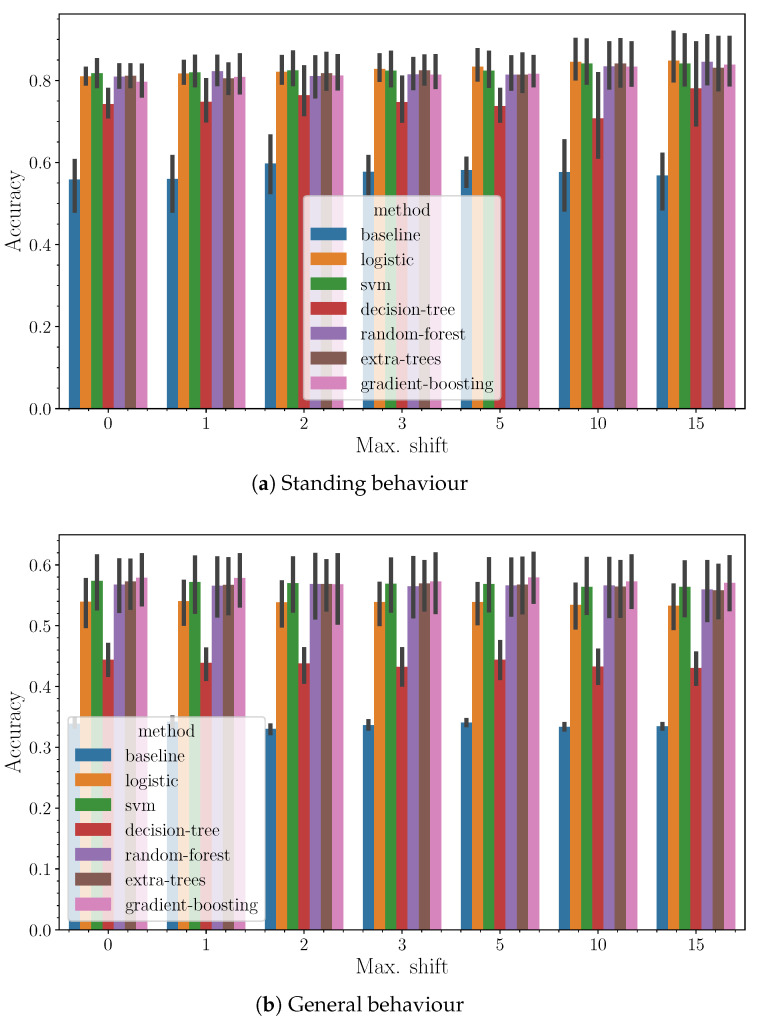
Accuracy (*y*-axis) as a function of the method (hue) and the maximum shift (*x*-axis) for a fixed window size of 120 s.

In Figure 7, we present detailed results highlighting how accuracy varies with the number of shifts, where a shift refers to steps incorporating information from the previous time window, with a fixed window size of 120 s. The data indicates a general trend: accuracy tends to improve slightly as the number of shifts increases. This suggests that the algorithms effectively leverage the incremental data from previous windows to enhance performance without falling into the trap of overfitting. Essentially, the addition of data points from successive time windows, each carrying less influential but still relevant information, appears to enrich the model’s learning process within the examined range of parameters. This trend underscores the algorithms’ capacity to assimilate and benefit from extended temporal data, optimizing accuracy while maintaining generalization capabilities.

The results for experiments conducted with a window size of 120 s and up to 10 shifts are compiled in Table 3 and Table 4. These tables also account for statistical uncertainty by including the standard error of the mean.

**Figure 8:**
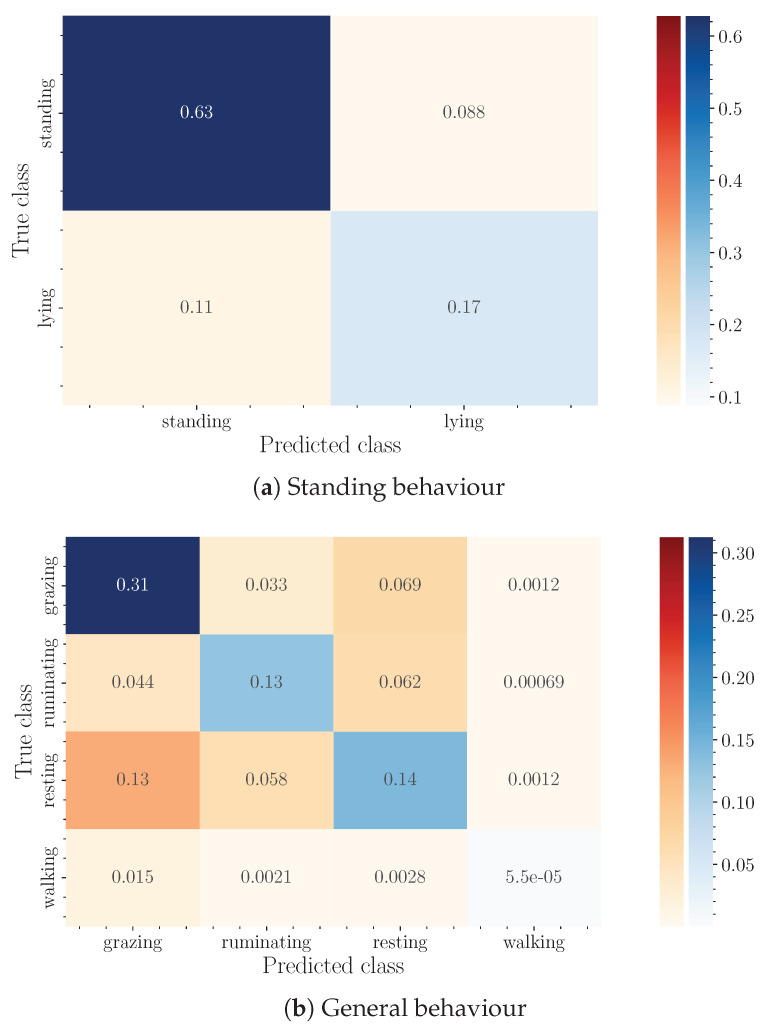
Confusion matrices for each of the groups of labels, normalized by the total observations.

Finally, confusion matrices for the best-performing algorithms for each of the groups of labels are shown in Figure 8. In these matrices, all instances have been added together and divided by the total number of instances to normalize the result. It should be noted that this does not match the average accuracy in each evaluation, which is the statistically correct estimator for the performance of the system when tested on a new cow. In the case of the first standing behavior, we have a binary classification with a mean accuracy of 0.85. Due to the uncertainty of the process and even of the labeling process itself, we can consider this result sufficiently satisfactory. In the case of the general behavior, we have a four-class classification with a negligible amount of instances in one of the classes. Among the remaining three, there is a confusion term between the grazing and resting classes, which are not so easily differentiated. Apart from that, the ruminating state is more clearly distinguished.

While it is true that there may be room for improvement in the results, such as through the study of hyperparameters of the models used, the selection of these is complicated because having data from only 9 groups would make it difficult to consider the statistics as sufficient. The results shown demonstrate the possibility of predicting the state of livestock using information from neck-mounted sensors up to a certain level of precision. Improving the limits of these metrics remains open as future work.

## 4. Conclusions

A system capable of detecting high-level features describing the condition of cattle has been defined and implemented. For this purpose, two groups of labels corresponding to two problems have been used: general behavior (4 classes) and standing behavior (2 classes). The accuracy achieved in these tasks is 0.57 and 0.85 respectively.

According to the study conducted, the generation of the most relevant features arises from the dispersion measurements of the time series of the sensors in the intervals in which the aggregation is performed. Means provide a smaller amount of information, while moments of order three provide a negligible amount of information.

The algorithms that produce the best models in the sense of accuracy are tree-based ensemble methods (random forest, gradient boosting, extra randomized trees) and support vector machines. The most computationally efficient algorithm with respect to the training time is extremely randomized trees in the cases with a small window size and support vector machines in those with a larger window size.

A direct comparison of these results with those from similar studies is always challenging since they are greatly dependent on the location of the sensor, the duration of the observations and the sets of behaviours to classify. In our case, the focus lies on the long-term monitoring of multiple animals in extensive farming, all factors that inevitably lead to an expected decrease in the performance of the algorithms. Indeed, the data labeling process is challenging in this setup, limiting the observer’s capabilities to correctly annotate the animal behaviour.

Despite this, we found our results consistent with those presented in the meta-analysis study in [7], which shows similar values of accuracy for standing and lying behaviour. The developed system can be used to detect lying bouts (transitions from standing to lying positions), which are a strong indicator for incoming calving events.

## Figures and Tables

**Table 1:** Labelling groups composition.

ID	Action
General behaviour	
A1	Grazing-Eating
A2	Ruminating
A3	Neutral
A4	Walking
Standing behaviour	
B1	Standing
B2	Lying

**Table 2:** Classifiers used. The short name is used below to identify the methods in the results section. The class name refers to the sklearn package.

Method	Short Name	Class	References
Baseline	baseline	dummy.DummyClassifier	-
Logistic regression	logistic	linear_model.LogisticRegression	[18]
Support vector regression	svm	svm.SVC	[19]
Decision tree	decision-tree	tree.DecisionTreeClassifier	[20]
Random Forest	random-forest	ensemble.RandomForestClassifier	[21]
Extra randomized trees	extra-trees	ensemble.ExtraTreesClassifier	[22]
Gradient boosting	gradient-boosting	ensemble.GradientBoostingClassifier	[23]

**Table 3:** Detailed metrics for Standing behaviour, using a 120 window size and a 10-step horizon.

Method	Time (s)	Accuracy	$F_{1}$	Precision	Recall	Cohen’s κ	Matthews’ φ
baseline	0.15 ± 0.02	0.58 ± 0.04	0.59 ± 0.06	0.63 ± 0.06	0.58 ± 0.04	−0.02 ± 0.01	−0.04 ± 0.02
logistic	0.17 ± 0.02	0.85 ± 0.03	0.84 ± 0.03	0.84 ± 0.02	0.85 ± 0.03	0.45 ± 0.09	0.46 ± 0.10
svm	0.58 ± 0.01	0.84 ± 0.03	0.83 ± 0.03	0.83 ± 0.03	0.84 ± 0.03	0.42 ± 0.10	0.43 ± 0.10
decision-tree	0.21 ± 0.02	0.71 ± 0.05	0.70 ± 0.06	0.74 ± 0.04	0.71 ± 0.05	0.17 ± 0.05	0.19 ± 0.05
random-forest	1.18 ± 0.05	0.83 ± 0.03	0.82 ± 0.03	0.82 ± 0.03	0.83 ± 0.03	0.39 ± 0.09	0.41 ± 0.09
extra-trees	0.55 ± 0.01	0.84 ± 0.03	0.83 ± 0.03	0.83 ± 0.03	0.84 ± 0.03	0.41 ± 0.10	0.43 ± 0.10
gradient-boosting	1.90 ± 0.11	0.83 ± 0.03	0.82 ± 0.03	0.82 ± 0.03	0.83 ± 0.03	0.39 ± 0.09	0.41 ± 0.09

**Table 4:** Detailed metrics for General behaviour, using a 120 window size and a 10-step horizon.

Method	Time (s)	Accuracy	$F_{1}$	Precision	Recall	Cohen’s κ	Matthews’ φ
baseline	0.69 ± 0.11	0.33 ± 0.00	0.34 ± 0.00	0.34 ± 0.01	0.33 ± 0.00	0.00 ± 0.00	−0.001 ± 0.003
logistic	1.66 ± 0.04	0.53 ± 0.02	0.52 ± 0.02	0.53 ± 0.02	0.53 ± 0.02	0.29 ± 0.02	0.30 ± 0.02
svm	71.00 ± 4.00	0.56 ± 0.02	0.55 ± 0.02	0.56 ± 0.02	0.56 ± 0.02	0.33 ± 0.03	0.34 ± 0.03
decision-tree	1.60 ± 0.09	0.43 ± 0.02	0.44 ± 0.02	0.45 ± 0.02	0.43 ± 0.02	0.16 ± 0.02	0.16 ± 0.02
random-forest	13.50 ± 0.50	0.57 ± 0.03	0.55 ± 0.02	0.56 ± 0.02	0.57 ± 0.03	0.33 ± 0.04	0.34 ± 0.04
extra-trees	4.87 ± 0.08	0.56 ± 0.02	0.55 ± 0.02	0.56 ± 0.02	0.56 ± 0.02	0.33 ± 0.03	0.33 ± 0.03
gradient-boosting	74.00 ± 3.00	0.57 ± 0.02	0.56 ± 0.02	0.56 ± 0.02	0.57 ± 0.02	0.34 ± 0.03	0.35 ± 0.03

## Data Availability

The data presented in this study were provided by González-Sánchez et al. [16].
